# Developing an Appropriate Evolutionary Baseline Model for the Study of Human Cytomegalovirus

**DOI:** 10.1093/gbe/evad059

**Published:** 2023-04-18

**Authors:** Abigail A Howell, John W Terbot, Vivak Soni, Parul Johri, Jeffrey D Jensen, Susanne P Pfeifer

**Affiliations:** School of Life Sciences, Center for Evolution and Medicine, Arizona State University, Tempe; School of Life Sciences, Center for Evolution and Medicine, Arizona State University, Tempe; Division of Biological Sciences, University of Montana, Missoula; School of Life Sciences, Center for Evolution and Medicine, Arizona State University, Tempe; School of Life Sciences, Center for Evolution and Medicine, Arizona State University, Tempe; School of Life Sciences, Center for Evolution and Medicine, Arizona State University, Tempe; School of Life Sciences, Center for Evolution and Medicine, Arizona State University, Tempe

**Keywords:** human cytomegalovirus, viral population genomics, evolutionary baseline model

## Abstract

Human cytomegalovirus (HCMV) represents a major threat to human health, contributing to both birth defects in neonates as well as organ transplant failure and opportunistic infections in immunocompromised individuals. HCMV exhibits considerable interhost and intrahost diversity, which likely influences the pathogenicity of the virus. Therefore, understanding the relative contributions of various evolutionary forces in shaping patterns of variation is of critical importance both mechanistically and clinically. Herein, we present the individual components of an evolutionary baseline model for HCMV, with a particular focus on congenital infections for the sake of illustration—including mutation and recombination rates, the distribution of fitness effects, infection dynamics, and compartmentalization—and describe the current state of knowledge of each. By building this baseline model, researchers will be able to better describe the range of possible evolutionary scenarios contributing to observed variation as well as improve power and reduce false-positive rates when scanning for adaptive mutations in the HCMV genome.

**Significance**

Human cytomegalovirus (HCMV) infection is a major cause of birth defects and can lead to severe effects in immunosuppressed and immunonaïve individuals. Pathogenicity is likely driven by multiple factors, including the genetic diversity of the virus itself. Furthermore, the accurate identification of genomic loci underlying viral adaptation relies on an appropriate baseline model that accounts for constantly operating evolutionary processes shaping this genetic diversity. With this overview of the current understanding of these processes in HCMV, we provide the necessary details for researchers to implement such a baseline model for their own genomic analysis of patient samples.

## Introduction

As the leading cause of infection-related birth defects—including cognitive and hearing impairments—human cytomegalovirus (HCMV) remains a major threat to global health, with a seroprevalence of more than 90% outside of the developed world (e.g., Boppana et al. 2013; Swanson and Schleiss 2013; Dreher et al. 2014). HCMV is also a primary cause of solid organ transplant failure (Balfour 1979) and often results in opportunistic infections in immunocompromised individuals or those with immature immune systems (e.g., Suárez et al. 2019, 2020). Additionally, primary infection or reactivation is implicated in a wide variety of health complications (Griffiths et al. 2015), and recent studies suggest that HCMV may play an active role in glioma pathogenesis in individuals with glioblastoma (Cobbs et al. 2002; Abdelaziz et al. 2019). Moreover, along with human immunodeficiency virus type 1 (HIV-1), HCMV is the most common viral agent transmitted from mother to offspring and may itself contribute to the vertical transmission of HIV-1 (Johnson et al. 2015; Girsch et al. 2022).

HCMV is a β-herpesvirus in the Herpesviridae family with a relatively large double-stranded (ds) DNA genome of ∼235 kb in size, including between 164 and 167 open reading frames (ORFs) (Dolan et al. 2004). Lytic infection is initiated by the expression of genes in a flow cascade, and DNA replication initiates 1–3 days postinfection (Weekes et al. 2014). The genome contains two unique regions—the unique long (U_L_) and unique short (U_S_) region—that are internally and externally flanked by repeats. The U_L_ region contains ORFs encoding gene products associated with latency and reactivation (Revello and Gerna 2010; Li et al. 2014); in laboratory passaged strains, cultures have been shown to accumulate large deletions in this region compared with clinically isolated viruses, likely owing to the relaxed selection in laboratory environments (Cha et al. 1996). In contrast, ORFs within the U_L_ region that encode envelope glycoproteins thought to be important for pathogenesis have been found to evolve under considerable constraint (He et al. 2006; Ji et al. 2006; Heo et al. 2008).

Multiple studies have suggested a link between pathogenesis and genomic variability (Meyer-König, Vogelberg, et al. 1998; Renzette et al. 2014; Wang et al. 2021), with high levels of diversity and multiple-strain infection found to be associated with higher viral loads (Pang et al. 2008; Sowmya and Madhavan 2009; Puchhammer-Stöckl and Görzer 2011). Furthermore, variation in the glycoproteins gO and gB, potentially generated through recombination (Meyer-König, Vogelberg, et al. 1998), has been proposed to influence cell tropism and dissemination (Hahn et al. 2004). Gaining a better understanding of the evolutionary forces that shape viral diversity is thus of critical importance both mechanistically and clinically. During the last decade, many efforts have been made to understand the relative contributions of admixture, positive and purifying selection, and infection-related bottlenecks in shaping HCMV interhost and intrahost variation (Renzette et al. 2013, 2015, 2017; Pokalyuk et al. 2017). Relatedly, numerous efforts have focused on elucidating key evolutionary parameters including the underlying mutation and recombination rates, as well as the selective effects of newly arising mutations (the distribution of fitness effects [DFE]; Renzette et al. 2015, 2017; Morales-Arce et al. 2022).

Importantly, recent studies focused upon evolutionary inference procedures have simultaneously demonstrated the value of jointly estimating parameters of natural selection with population history, as a neglect of one to infer the other will often result in serious misinference (Johri et al. 2020, 2021). Moreover, only by first accounting for the constantly acting evolutionary processes of genetic drift (as shaped by the infection bottleneck and subsequent viral population growth, as well as the genetic structure associated with compartmentalization) and purifying and background selection (owing to the pervasive input of deleterious mutations) may one develop a meaningful baseline model of expected levels and patterns of genomic variation. This baseline model is critical for accurately detecting and quantifying rarer and episodic evolutionary processes, such as positive selection potentially leading to viral adaptation (Johri, Aquadro, et al. 2022; Johri, Eyre-Walker, et al. 2022). More specifically, owing to overlapping patterns between neutral and selective evolutionary processes (Jensen 2009; Bank et al. 2014), this baseline model is essential for defining rates of true positives and false positives associated with the detection of rare or episodic effects in any given population and for any given data set.

As such an evolutionary baseline model has yet to be fully described for HCMV, we here outline important components of such a model and review the current state of knowledge pertaining to each: mutation rates, recombination rates, the distribution of fitness effects, infection dynamics, and compartmentalization. We close with a series of recommendations for improving evolutionary inference in this important human pathogen and highlight key areas in need of further investigation.

## Mutation Rate

The mutation rate quantifies the frequency at which spontaneous (*de novo*) mutations arise in a genome, as caused by a variety of factors including DNA replication errors and spontaneous DNA damage (see review of Pfeifer 2020). This rate is distinct from the substitution rate—that is, the rate at which mutations become fixed in a population—which is influenced not only by the *de novo* mutation rate but also by natural selection, genetic drift, as well as multiple other factors. However, for strictly neutral mutations, the rate of mutational input is equal to the rate of substitution (Kimura 1968), leading to a clock-like accumulation of mutations over time. Using a molecular clock (divergence)-based approach, recent studies have reported substitution rates of approximately 3.0 × 10^−9^ substitutions per nucleotide per year in HCMV (McGeoch et al. 2000)—one to two orders of magnitudes lower than the rate reported for a closely related virus, herpes simplex virus (HSV-1), which exhibits 3.0 × 10^−8^ (Sakaoka et al. 1994) and 1.4 × 10^−7^ (Kolb et al. 2013) substitutions per nucleotide per year. Mutation rates of both HCMV and HSV-1 have also been studied *in vitro*. For example, by scoring null mutations in the *tk* gene using ganciclovir, mutation rates in HSV-1 have similarly been estimated to range from 5.9 × 10^−8^ (Hwang et al. 2002; Drake and Hwang 2005) to 1.0 × 10^−7^ (Hall and Almy 1982) substitutions per nucleotide per cell infection, where cell infection is an estimate of a viral generation.

It is necessary here to highlight the various units being reported when comparing between the results described in different studies, with rates reported as substitutions per nucleotide per generation (s/n/g), substitutions per nucleotide per year (s/n/y), substitutions per nucleotide per cell infection (s/n/c), or substitutions per nucleotide per round of copying (s/n/r), if the mode of replication is known. The mode of replication of dsDNA viruses is likely limited to semiconservative replication, although RNA viruses by comparison are known to use a “stamping machine” model, where a single template is used for all progeny strands (Luria 1951). To compare between estimates using substitutions per nucleotide per cell infection and estimates using substitutions per nucleotide per year, we have used the number of viral cycles per year as a conversion factor (table 1). Specifically, conversion factors of 181.87 to 362.48 viral cycles per year were chosen to span lower and upper estimates for HCMV, while 1,946.67 viral cycles per year were used for closely related HSV-1 for comparison. These estimates are based on internalization times of 10 min (Bodaghi et al. 1999; Hetzenecker et al. 2016) and 30 min (Zheng et al. 2014), as well as eclipse times of 24–48 h (Jean et al. 1978) and 4 h (Nishide et al. 2019), for HCMV and HSV-1, respectively. Importantly, these conversions highlight the discrepancy between divergence and *in vitro* estimates of the substitution rate, demonstrating that molecular clock-based estimates primarily provide information about the rate of neutral and nearly neutral mutation, rather than estimating full mutational spectra (as discussed in the below section). Additionally, the further analysis of future patient samples would be of great value in better characterizing the interhost variance in these rates.

**Table 1:** *In Vitro-* and Divergence-Based Estimates of *De Novo* Mutation Rates in HCMV Compared with the Closely Related HSV-1

Virus	Approach	Original Unit^a^	Estimated Rate/Cycle	Reference
HCMV	*In vitro*	s/n/c	2.0 × 10^−7^	Renzette et al. 2015
HCMV	Divergence	s/n/y	1.6 × 10^−11^ / 8.2 × 10^−12^	McGeoch et al. 2000
HSV-1	Divergence	s/n/y	7.1 × 10^−11^	Kolb et al. 2013
HSV-1	Divergence	s/n/y	4.1 × 10^−11^	Sakaoka et al. 1994
HSV-1	*In vitro*	s/n/c	1.0 × 10^−7^	Hall and Almy 1982
HSV-1	*In vitro*	s/n/c	5.9 × 10^−8^	Hwang et al. 2002; Drake and Hwang 2005

Note.—To compare between estimates using substitutions per nucleotide per cell infection (s/n/c) and estimates using substitutions per nucleotide per year (s/n/y), we have used conversion factors of either 181.87 or 362.48 viral cycles per year to span uncertainty in HCMV, and 1,946.67 viral cycles per year for HSV-1.

a s = substitutions; n = nucleotide; c = cell infection; y = year.

Notably, these experimental and empirical measurements of the mutation rate based on genome-wide population genetic data neglect the substantial proportion of lethal and deleterious mutations that are removed from the population via purifying selection. Owing to this neglect, measurements obtained using these methods are likely an underestimate of the genuine genome-wide mutation rate (Peck and Lauring 2018). Mutation accumulation experiments provide a valuable (and less biased) alternative by subjecting a viral population to a series of bottlenecks that reduces the effective population size, thus minimizing the efficacy of selection. A similar strategy can be applied to natural, longitudinal population data. Using this approach, the mutation rate of HCMV was estimated by Renzette et al. (2015) as 2.0 × 10^−7^ mutations per nucleotide per generation using longitudinal samples obtained from 18 patients, where mutations were called if absent in earlier samples and present in all later samples. Importantly, however, evaluating such longitudinal data in the context of a mutation accumulation study comes with the qualification that selective pressures are expected to be much stronger in patient samples relative to traditional experimental mutation accumulation lines. In addition, the presence of a reinfection event during the longitudinal sampling—if not identified—would be expected to upwardly bias these estimates. It is also important to note that rate estimates of this sort are further complicated by practical limitations of clinical sampling. Specifically, previous studies have shown that deep sequencing through the use of polymerase chain reaction amplicons requires rare variants to be present at >1% frequency in order to be reliably detected (Fonager et al. 2015; Kyeyune et al. 2016)—though newer methods that utilize target enrichment protocols may improve upon this threshold (Hage et al. 2017). Given that the vast majority of variants are expected to be rare, such detection thresholds may be of considerable significance.

Mutation rates in viruses may evolve through both mutator and antimutator alleles, the fixations of which are thought to be governed by genome size and effective population size (Lynch et al. 2016). When effective population sizes are small, selection is weak and may be unable to prevent mutator alleles from fixing. To date, one hypermutator has been identified in HCMV (Chou et al. 2016). Mutator alleles are a double-edged sword for viruses, having important implications for the rate of adaption (Taddei et al. 1997; Travis and Travis 2002), but more significantly also create the possibility of mutational meltdown (Crotty et al. 2001; Beaucourt et al. 2011; Bank et al. 2016; Matuszewski et al. 2017; Ormond et al. 2017). Indeed, owing to interference between the greater input of deleterious mutations with the minor input of beneficial mutations, higher mutation rates may slow or stop the rate of adaptation (Pénisson et al. 2017; Jensen and Lynch 2020; Jensen et al. 2020). Other molecular determinants of viral mutation rates include postreplicative repair through interaction with DNA damage response pathways (Weitzman et al. 2010; Luftig 2014)—a particularly relevant mechanism for HCMV as herpesviruses are known to induce DNA damage responses (Xiaofei and Kowalik 2014).

As HCMV has been observed to be quite diverse compared with other DNA viruses—on the order of certain RNA viruses (Wang et al. 2002; Jerzak et al. 2005)—one formal possible explanation for the high levels of nucleotide diversity observed in HCMV is an exceptionally high mutation rate (i.e., as levels of neutral variation are expected to be a factor of the effective population size as well as the underlying mutation rate). This hypothesis was recognized as unlikely by Renzette et al. (2011), owing, among other reasons, to the proofreading activity of HCMV's DNA polymerase (Nishiyama et al. 1983). Although Cudini et al. (2019) recently rediscussed this possibility (and see the response of Jensen and Kowalik 2020), there appears to be general agreement that RNA virus-like levels of variation in HCMV are not due to RNA virus-like mutation rates. Specifically, following multiple studies on HCMV interhost and intrahost variation (Renzette et al. 2013, 2015, 2017; Pokalyuk et al. 2017; and see the below sections), it has been demonstrated that observed diversity is likely generated by a combination of mutation, recombination, reinfection, compartmentalization, selection, and infection population size histories (Jensen 2021)—with a mutation rate of 2.0 × 10^−7^ mutations per nucleotide per generation appearing consistent with the data (Renzette et al. 2015). More specifically, the observed high levels of variation appear to more likely be related to the population dynamics related to compartmentalization, gene flow, and reinfection, rather than to particularly elevated rates of mutation (e.g., Pokalyuk et al. 2017; Jensen and Kowalik 2020). Renzette et al. additionally identified a weak but highly significant positive correlation between estimated mutation rates and single nucleotide polymorphism (SNP) density across the HCMV genome, as may be expected. Heterogeneity in mutation rates across the genome was additionally proposed as a contributing factor underlying the observed correlations between intraspecies variation and recombination rates, as well as of that between variation and divergence (Renzette et al. 2016).

## Recombination Rate

Recombination not only contributes genetic variation through the generation of novel genotypic combinations, but it may also improve the efficacy of selection through the reduction of interference effects between and among beneficial and deleterious variants (Hill and Robertson 1966; Felsenstein 1974; Lynch et al. 1995; Pénisson et al. 2017). Studies examining the intergenic variability of HCMV glycoprotein loci (Meyer-König, Haberland, et al. 1998; Haberland et al. 1999; Yan et al. 2008) provided the initial evidence for homologous recombination in the HCMV genome. Nearly two decades later, Renzette et al. (2015) estimated a genome-wide recombination map using a population genetic approach, reporting a mean recombination rate of ∼0.23 crossover events per genome per generation, based on observed patterns of linkage disequilibrium (LD) (i.e., by assessing the extent to which observed haplotype distributions may be explained by variable rates of recombination; and see the review of Stumpf and McVean (2003) for a discussion on estimating recombination rates from population genetic data). The authors further reported a correlation between recombination rate and SNP density, consistent with widespread purifying selection, as has been observed in multiple diverse species (e.g., Begun and Aquadro 1992; Pfeifer and Jensen 2016; Renzette et al. 2017; and see the review of Charlesworth and Jensen 2021). However, as with mutation rates, recombination rate estimates can also be misinferred, for example, due to unaccounted for progeny skew, which is known to increase levels of LD in highly skewed populations relative to standard Wright–Fisher expectations (and as such may downwardly bias recombination rate estimation if unaccounted for; Eldon and Wakeley 2008; Birkner et al. 2013). This observation highlights the need for further computational method development of mutation and recombination rate estimators for the type of generalized progeny skew distributions applicable to viruses and other human pathogens (Morales-Arce et al. 2020; Sabin et al. 2022).

In addition to LD–based approaches, studies have also characterized recombination in the HCMV genome using a combination of phylogenetic and population-level analyses. By constructing “phylogenetic trees” for each gene in the HCMV genome and correcting for recombination breakpoints with the genetic algorithm GARD, Kosakovsky Pond et al. (2006) found that the majority of loci showed no consistent phylogenetic patterns, indicating that recombination occurs often enough that whole genomes can behave as “gene-scale mosaics.” In other words, what certain authors refer to as variable phylogenetic trees are in fact better described as variable coalescent histories. Further, like the Renzette et al. studies, Sijmons et al. (2015) also observed a correlation between recombination rate and nucleotide diversity using a phylogenetic approach. However, phylogenetic-based approaches are generally poorly suited for the study of recombination compared with the coalescent-based approaches utilized in population genetics—and multiple studies suffer from these limitations when trying to distinguish between recombination and competing evolutionary processes in a phylogenetic framework (e.g., Houldcroft et al. 2016; Cudini et al. 2019). Specifically, coalescent theory provides a sophisticated framework for the study of variable gene genealogies owing to recombination (Wakeley 2009) and avoids the pretense of searching for a single (and nonexistent) “phylogenetic tree’ to describe within-population variation (e.g., Cudini et al. 2019; and see Rosenberg and Nordborg 2002 for a discussion).

## The Distribution of Fitness Effects (DFE)

HCMV is characterized by a large genome relative to other human viruses. Although the set of protein-coding genes in HCMV experiences constant revision, there are 45 core genes that are conserved across all herpesviruses and ∼117 noncore genes that are more specific to the CMVs, many of which are still being functionally characterized (Van Damme and Van Loock 2014; Mozzi et al. 2020). Although it is clear that protein-coding regions occupy the majority of the HCMV genome, these uncertainties mean that the precise fraction of the genome that experiences direct purifying selection is not yet fully defined—though roughly 25% of the genome has been observed to be nearly devoid of variation, potentially suggesting strong constraint (Renzette et al. 2015). Interestingly, within-patient nucleotide diversity in noncoding regions of the genome has generally been observed to be on the same order as less-constrained coding regions (Renzette et al. 2011), suggesting the presence of functionally important regions interspersed across the genome and/or widespread background selection effects (Renzette et al. 2016). This combination of factors renders the identification of neutrally evolving sites challenging.

Previous studies have used comparisons of sequence evolution at nonsynonymous versus synonymous sites at various evolutionary scales to quantify selective forces acting on protein-coding regions in the HCMV genome. A comparative genomic analysis across multiple CMV species found pervasive purifying selection in most protein-coding regions (as indicated by low levels of *d*_N_/*d*_S_; Mozzi et al. 2020), as would be expected. Similarly, comparisons of sequence polymorphism within hosts to the divergence among hosts (i.e., using the McDonald and Kreitman 1991 test) also indicated the action of widespread purifying selection (Renzette et al. 2011). In contrast, evidence for positive selection was limited to specific regions, including the glycoproteins (Renzette et al. 2013). Thus, although glycoproteins and their linked regions will likely be additionally impacted by recurrent selective sweeps, the majority of the genome is expected to be largely affected by the direct and linked effects of purifying selection.

**Fig. 1.**
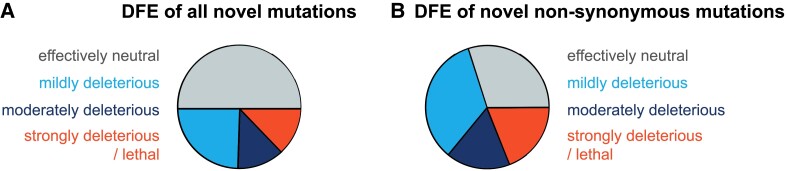
Distribution of fitness effects (DFE) of all new and new nonsynonymous mutations. (*A*) Using an approximate Bayesian framework to account for the specific demographic history of their within-patient population, Morales-Arce et al. (2022) inferred the DFE of all new mutations in human cytomegalovirus as roughly 50% effectively neutral ($-1<2N_{e}s\leq 0$ ; gray), 24% mildly deleterious ($-10<2N_{e}s\leq -1$ ; light blue), 12% moderately deleterious ($-100<2N_{e}s\leq -10$ ; dark blue), and 13% strongly deleterious/lethal ($2N_{e}s\leq -100$ ; red), where $N_{e}$ refers to the effective population size and *s* to the selection coefficient against the homozygote. (*B*) Assuming that ∼30% of all sites in coding regions likely have little or no fitness costs upon mutation, the DFE at functionally important sites corresponds to roughly 30% effectively neutral, 34% mildly deleterious, 17% moderately deleterious, and 19% strongly deleterious/lethal mutations.

As selection against harmful mutations at functionally important sites in the genome can affect patterns of variation at linked neutral alleles (i.e., background selection; Charlesworth et al. 1993) and as this effect has been suggested to be a primary determinant of genomic variation in HCMV (Renzette et al. 2016), it is important to characterize the DFE of newly arising mutations across the genome. A recent study by Morales-Arce et al. (2022) used an approximate Bayesian computation (ABC) framework to infer the DFE of deleterious mutations from a within-patient sample of HCMV. This study accounted for the specific demographic history of the within-patient population as associated with viral infection dynamics (as previously inferred by Renzette et al. 2013), non-Wright–Fisher replication dynamics, as well as background selection. They inferred that roughly 50% of all new mutations were effectively neutral ($-1\;<\;2N_{e}s\leq 0$), 24% were mildly deleterious ($-10\;<\;2N_{e}s\leq -1$), 12% were moderately deleterious ($-100\;<\;2N_{e}s\leq -10$), and 13% were strongly deleterious ($2N_{e}s\leq -100$), where $N_{e}$ refers to the effective population size and *s* to the selection coefficient against the homozygote (fig. 1*A*). As these estimates were obtained for all sites comprising the functional region (i.e., the inference was not restricted to nonsynonymous sites) and ∼30% of all sites in coding regions are likely to have little or no fitness costs upon mutation (e.g., synonymous changes), the DFE at functionally important sites in HCMV is probably closer to 30% effectively neutral, 34% weakly deleterious, 17% moderately deleterious, and 19% lethal mutations (fig. 1*B*). Importantly, although such a correction naturally depends on the fraction of synonymous sites that are behaving neutrally, these estimates are in fact quite consistent with multiple previous random mutagenesis studies that measured the proportion of lethal mutations in DNA viruses to be ∼20% (e.g., Sanjuán 2010). While Morales-Arce et al. (2022) accounted for a number of factors that add complexity to within-patient populations of HCMV (including an extremely strong bottleneck corresponding to the infection), they simulated only a single population of HCMV. As there is strong evidence of HCMV populations being structured within patients (Pokalyuk et al. 2017; Sackman et al. 2018; and see the section on Compartmentalization below), current estimates of the deleterious DFE might still be biased, and future inference incorporating both compartmentalization and reinfection will be important in this regard.

## Infection Dynamics

The demographic history of a population is an important determinant of both genetic variation and potential selective outcomes and therefore an appropriate starting point for evolutionary analysis, particularly in light of the high levels of HCMV diversity observed within patients (Drew et al. 1984; Spector et al. 1984; Haberland et al. 1999; Faure-Della Corte 2010; Renzette et al. 2011, 2013, 2015, 2016, 2017; Hage et al. 2017; Pokalyuk et al. 2017). The expected intrahost population dynamics involve a strong population bottleneck (a temporary reduction in population size) at the point of infection, followed by rapid population expansion (see review of Jensen 2021). The level of intrahost genetic variation that is present at the point of infection will in part be determined by the severity of the bottleneck. If the transmission bottleneck is wide, then there may be numerous virions founding the initial infection, resulting in greater genetic variation and an increased probability that beneficial variants may be transferred from the founding population. Conversely, a narrow bottleneck can result in a severe loss of genetic variation, with low-frequency variants being eliminated regardless of their fitness effects. This process is known as a founder effect (see Zwart and Elena 2015, for a discussion of this effect in viral populations).

**Fig. 2.**
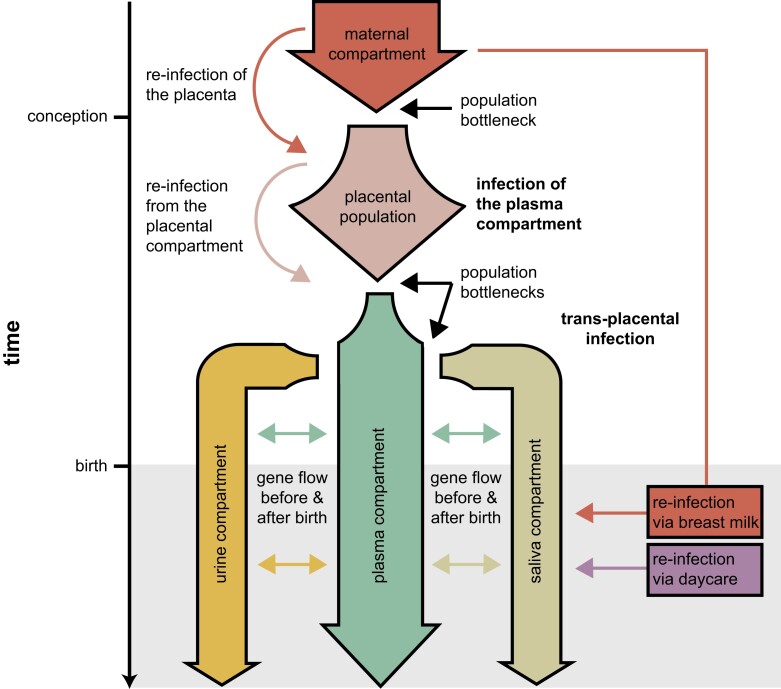
Demographic dynamics of congenital human cytomegalovirus (HCMV) infection. Demographic scenarios of infection and reinfection in HCMV likely contributing to the high levels of observed interhost and intrahost diversity, including a population bottleneck associated with the initial transplacental infection (transmission of virions from the maternal compartment [red]/plasma [pink] to the fetal plasma compartment [green]), followed by additional bottlenecks associated with compartmental infections (urine [yellow] and saliva [olive]), as well as gene flow between compartments and reinfection of compartments during pregnancy and after birth (e.g., via breast milk [red] and/or daycare [purple]).

In the case of congenital infections, demographic modeling approaches have shown support for a population bottleneck associated with the initial transplacental infection (transmission of virions from the maternal compartment to the fetal plasma compartment), followed by additional bottlenecks associated with compartmental infections (fig. 2; and Renzette et al. 2013; for a detailed discussion regarding the population structure dynamics between compartments, see the section below). Importantly, the initial bottleneck was shown to involve potentially hundreds of unique HCMV genomes, which helps to explain the relatively high levels of genetic diversity observed at the point of infection, as compared with certain RNA viruses in which a single (or very few) virions are thought to be involved in infection (Keele et al. 2008; Fischer et al. 2010; Renzette et al. 2013, 2014). Furthermore, Renzette et al. (2013) found support for gene flow between urine and plasma compartments (the two compartments sampled in that study). Their results further suggested that plasma may serve as a “route” for gene flow within the host, with preliminary evidence indicating that it carries compartment-specific variants from other compartments; this process may thus also be an important determinant of within-host variation.

Further evidence for admixture between compartments (this time including plasma, urine, and saliva compartments) was found by Pokalyuk et al. (2017), suggesting that reinfection postbirth is possible via, for instance, breast milk (Numazaki 1997; Enders et al. 2011; also see the review of Bardanzellu et al. 2019). In other words, maternal compartment-specific variants appeared to be transmitted to the infant postbirth. Although the above examples are focused upon congenital infections, related work has similarly highlighted the importance of multistrain infections in immunosuppressed adults and particularly the relationship between this infection status and the emergence of antiviral resistance mutations (e.g., in transplant recipients; Suárez et al. 2019, 2020).

To date no method exists to prevent maternal–fetal transmission or to reduce the severity of fetal infection (Britt 2017). Therefore, the characterization of population dynamics is likely to be integral to future therapeutic strategies. For example, clinically imposing a more severe population bottleneck during pregnancy may reduce genetic variation in the HCMV infecting population, limiting the pool of variation on which natural selection may subsequently act, thereby potentially improving treatment outcomes. Finally, it has been shown that host immune suppression can reactivate dormant viruses, restarting production of viral progeny; this switch from latent to productive life cycles can induce temporary or sustained CMV replication (Porter et al. 1985; Dupont and Reeves 2016).

Demographic inference in HCMV is inherently challenging due to the genome-wide impact of selection (see the DFE section above), which will in turn bias common demographic estimators which are based on neutrality (see the discussion of Ewing and Jensen 2016; Pouyet et al. 2018). Namely, neutral demographic estimators require sufficiently large nonfunctional regions and high rates of recombination, such that assumptions of strict neutrality hold (Gutenkunst et al. 2009; Excoffier et al. 2013; Kelleher et al. 2019; Steinrücken et al. 2019). These criteria ensure that variants can be chosen that are not experiencing background selection. For example, Renzette et al. (2013) utilized *∂a∂i*, a neutral demographic inference approach based on the site frequency spectrum (Gutenkunst et al. 2009), to build and parameterize HCMV infection models (and see Sackman et al. 2018; Jensen and Kowalik 2020).

This inference problem of estimating demography in the presence of selection is indeed somewhat circular, as the estimation of selection will also be biased by unaccounted for demographic dynamics (Rousselle et al. 2018; Johri et al. 2020). This fact highlights the importance of performing joint, simultaneous inference of selection with demography, rather than taking the more common stepwise approach of first estimating one and then the other (see review of Johri, Eyre-Walker, et al. 2022). Recently proposed ABC approaches that jointly estimate population history and the DFE of deleterious mutations perform such joint inference and importantly do not require the *a priori* identification of neutrally evolving sites (Johri et al. 2020). Explicitly accounting for viral infection dynamics, Morales-Arce et al. (2020) incorporated progeny skew into the joint ABC inference scheme of Johri et al. (2020)—an important extension to this framework as the assumption of small progeny distributions utilized by a majority of population genetic inference approaches is likely violated in many pathogens, as noted above (see reviews of Tellier and Lemaire 2014; Irwin et al. 2016). The authors demonstrated that their tailoring of this ABC inference approach specifically to viral populations avoided misinference resulting from a neglect of this consideration. Other recent inference approaches have also relaxed the assumption of small progeny skew, demonstrating an ability to coestimate parameters related to the biology of progeny skew together with those of demographic and selective histories (e.g., Matuszewski et al. 2018; Sackman et al. 2019).

## Compartmentalization

The final consideration of note impacting intrahost population dynamics of viral infections is population structure between different areas of infection, commonly referred to as compartmentalization (Zárate et al. 2007). Compartmentalization may be relevant for any virus not localized to a single organ or cell type (Di Liberto et al. 2006; Zárate et al. 2007; Renzette et al. 2014; Sackman et al. 2018)—including HCMV, known to infect several cells and organs throughout the body.

As a long-studied virus, HCMV has been well documented to infect a wide variety of cells including the epithelial cells of gland and mucosal tissue, smooth muscle cells, fibroblasts, macrophages, dendritic cells, hepatocytes, and vascular endothelial cells (Sinzger et al. 2008; Jean Beltran and Cristea 2014). Unsurprisingly given this broad cellular tropism, evidence of infection in specific organs is similarly extensive and includes the brain and peripheral nerves, the eyes, the placenta, the lungs, the gastrointestinal tract from the esophagus to the colon, the liver, the lymph nodes, the heart, the peripheral blood, and the kidneys (Plachter et al. 1996). Of these areas, viral shedding from salivary glands, the ductal epithelium of mammary glands and the kidney, and the syncytiotrophoblasts (placenta) is thought to be critical to interhost transmission (Mocarski 2004; Kinzler and Compton 2005). However, because of potential gene flow between compartments within a host, other sites of infection are nonetheless important for understanding the intrahost dynamics of this virus.

Another necessary consideration is the location of regions that can harbor the latent stage—these areas are likely important for the maintenance of genetic diversity that may otherwise be lost in actively replicating lineages (Chou 1989; Frange et al. 2013). While infections can occur across the body, the latent, and importantly nonreproducing, stage of the virus seems to be limited in cell tropism. Specifically, HCMV has been found to use endothelial and select myeloid lineages as well as monocytes, macrophages, and their progenitors (i.e., cells found in the circulating plasma population) as latency sites (Jarvis and Nelson 2002; Yatim and Albert 2011).

Given the wide range of potential sites of infection, it is crucial to resolve observed levels of intrahost population structuring that are indicative of compartmentalization. Several studies have observed considerable genomic diversity (Renzette et al. 2011, 2013; Mayer et al. 2017; Pokalyuk et al. 2017; Cudini et al. 2019; Pang et al. 2020), while others have found intrahost populations to be comparatively invariant (Hage et al. 2017). The comparison of patients with single- versus multiple-infection histories is likely one important source of disparity in these observed levels of variation (Mayer et al. 2017; Pokalyuk et al. 2017; Sackman et al. 2018; Cudini et al. 2019; Jensen and Kowalik 2020; Houldcroft et al. 2020; Pang et al. 2020). It should also be noted that the importance of multiple infections in shaping intrahost diversity of infants may still rely on compartmentalization within the maternal infection (e.g., with primary infections arising from the cervical population and secondary infections being associated with the mammary gland population; Sackman et al. 2018; Pang et al. 2020).

Compartmentalization has also been implicated as a clinically important factor in the development of a multidrug resistant lineage within the chronic infections of immunocompromised patients (Frange et al. 2013; Renzette et al. 2014; Suárez et al. 2019, 2020). Furthermore, multiple population genetic studies using longitudinally sampled patient data concluded that compartmentalization is an important factor in explaining intrahost diversity of fetal and infant infections (Renzette et al. 2013, 2015). Models developed from these studies focused on three subpopulations corresponding to source sites of samples: salivary glands/saliva, blood/plasma, and kidney/urine (Renzette et al. 2014, 2015; Pokalyuk et al. 2017; Sackman et al. 2018). Generally, these models attribute plasma as the circulating population that serves as an intermediary for spread between the distal compartments of salivary glands and kidney (fig. 2). Of particular note, levels of genetic divergence between compartments of a single patient were found to be as great as those observed between the same compartment sampled from unrelated patients (Renzette et al. 2013), suggesting limited between-compartment gene flow within a single host. Yet, the extent to which these considerable levels of differentiation are attributable to localized, compartment-specific adaptation, or simply the constant operation of neutral evolutionary processes, remains unresolved—and this continues to stand as one of the most pressing and interesting evolutionary questions in the HCMV system.

## Closing Thoughts

When developing an evolutionary baseline model of HCMV, special consideration should be given to the demographic processes that shape genetic diversity and the sampling methods that generate clinical data sets, including the ability to detect low-frequency variants, as well as the level of progeny skew, bottleneck severity during infection and reinfection, and the degree of compartmental admixture. Correctly modeling these processes and accounting for various ascertainment biases will allow researchers to better describe the relative contributions of each evolutionary force in shaping observed levels and patterns of variation, as well as quantify uncertainty in model choice and in the identification of adaptive loci. In addition, gaining a better understanding of when and how HCMV diversity is generated has important implications for vaccine development as well as antiviral therapy, both for determining the timing of drug delivery and for combating resistance evolution.
